# Supplementary material for: Are carbon emissions trading and green financial instruments synergistic? -Comprehensive quantitative research based on content analysis
Source: PLoS One. 2024 Mar 7;19(3):e0298601. doi: 10.1371/journal.pone.0298601 (PMC10919601; doi:10.1371/journal.pone.0298601)
Supplement: S1 Data — (ZIP) [file pone.0298601.s001.zip › Data/地级市Z_F工作报告.zip!/║■▒▒/╛ú╓▌╩╨/└┤╫╘╣1⁄2╓┌║┼í╛┬φ┐╦╩2╛▌═°í┐_2006.docx]

各位代表：

现在，我代表市人民政府向大会作工作报告，请连同《荆州市经济和社会发展第十一个五年规划纲要（草案）》一并审议，并请市政协各位委员和其他列席人员提出意见。

一、“十五”时期经济社会发展及2005年工作回顾

“十五”时期，在省委、省政府和市委的正确领导下，在市人大、市政协的监督支持下，市政府坚持以邓小平理论和“三个代表”重要思想为指导，认真贯彻和落实科学发展观，深入推进改革开放，主动适应国家宏观调控政策，克服“非典”等疫情和各种自然灾害造成的不利影响，实现了经济持续快速增长。全市生产总值年均增长8.5%，比“九五”时期高0.4个百分点。社会消费品零售总额达到221.6亿元，年均增长10.7%。外贸出口累计7.3亿美元，年均增长23.4%。财政收入达到24.4亿元，年均可比增长9.6%。

--全面实施“工业兴市”战略，深化企业产权制度改革，大力推进重点行业发展和园区建设，加快了产业结构调整和工业化进程。以企业产权多元化和转换职工身份为重点，扎实开展企业改革改制工作，全市国有工业企业改制面达90%以上，市直工业企业改革任务基本完成。规模以上工业企业五年新增92家，工业增加值年均增长11.1%，汽车零部件、纺织、家电、化工、森工造纸、食品加工等行业产能成倍增长，工业整体素质明显提高。荆州工业新区一期工程顺利建成，开发区、工业园区发展势头良好，工业发展平台初具规模。

--全面开展农村税费改革，认真落实扶农惠农政策，积极调整农业结构，农业综合生产能力提高，农民收入明显增加。减免农业税费和发放补贴资金，切实减轻农民负担，农民普遍得到较大实惠。推进农村综合配套改革和乡镇机构改革，减少财政供养人员，转变基层政权管理职能，干群关系进一步密切。农民人均纯收入达到3108元，比2000年增加923元，年均增长7.3%。

--全面扩大投资建设规模，基础设施支撑能力整体提升，增强了可持续发展能力。全社会固定资产投资累计完成512.4亿元，突破500亿元大关，年均突破100亿元，比“九五”时期增加209.2亿元，年均增长7.5%。交通累计投资63亿元，是“九五”时期的3倍；荆州长江大桥和沪蓉、荆襄高速公路两个立交互通区建成通车，开工建设荆东高速公路、随岳高速公路荆州段，“一横一纵”高速公路骨架初步形成；新改建等级以上公路3150公里，基本实现了乡乡通油路、村村通公路。水利累计投资43亿元，相当于2000年前50年的投资总和，重点实施荆江大堤和长江干堤整险加固工程，长期困扰荆州发展的洪水威胁得到有效治理。电力、电信累计投资分别为18.5亿元、18亿元，比“九五”时期增加3.6亿元、2.1亿元，对经济社会发展的保障能力进一步增强。

--全面加快城市化进程，城市集聚辐射功能不断增强，城乡面貌发生可喜变化。加强城市规划、建设和管理，积极开展城市资本营运，实行商业用地招、拍、挂制度，规范房地产市场，改善城市居民住房条件和环境，解决了一批关系群众生产生活的突出问题。努力争取开发银行贷款，配套完善城市道路设施。开展渍水改造工程，启动护城河、西干渠整治等水环境综合治理工程，城市基础设施条件明显改善。中心城区累计完成基础设施投资5.4亿元，建成区扩大5.2平方公里，区域性龙头带动作用进一步发挥。加强县市城区和7个国家级、10个省级重点镇建设，全市城市化率达到35%，比2000年提高2个百分点。

--全面推进和谐社会建设，坚持依法行政，精神文明和民主法制建设得到加强，社会保障、教育、卫生等社会事业发展成绩很大，人民群众生活水平进一步提高。社会保障覆盖面不断扩大，社保费收入达9.9亿元，比2000年增加4.6亿元，年均增长14%。累计发放国有企业下岗职工基本生活费、离退休人员养老金和失业保险金分别为2.2亿元、36.4亿元和2.5亿元；累计发放城镇低保资金3.7亿元，解决困难群众基本生活问题。就业状况逐步改善，累计新增城镇就业31.4万人，年均增长9%。实行以县为主的农村义务教育管理体制，义务教育预算内经费达到6.8亿元，比2000年增加4亿元，年均增长19.4%。市县疾病预防控制和医疗救治体系基本建立，血吸虫病综合防治取得新进展。高度重视人口、资源和环境保护工作，人口出生率控制在8‰以内。积极发展文化、体育事业，加强社区建设，推动精神文明创建活动深入开展。加强社会治安综合治理和安全生产管理，积极化解各类矛盾，社会秩序保持稳定。努力改善人民生活，城镇居民人均可支配收入达到8094元，年均增长9.1%；城乡居民储蓄存款余额达到345.9亿元，年均增长20.3%。

2005年是“十五”最后一年，全市上下以保持共产党员先进性教育活动为动力，深入推进“工业兴市”战略，促进经济社会协调发展，较好地落实了年初预期目标任务。地区生产总值增长8.1％，其中：一、二、三产业分别增长3%、10.9%和9.7%。固定资产投资增长7.9%，财政收入可比增长8.4％，社会消费品零售总额增长12.5％，外贸出口增长28％，城镇居民人均可支配收入增长6.5％，农民人均纯收入增长3.5％。

（一）国民经济平稳较快发展。工业发展步伐进一步加快。规模以上工业企业达到572家、新增33家，完成增加值82亿元、增长17.8%，是自1998年开始统计该项指标7年来的最好水平。高新技术产业增加值22亿元，增长14.6%。工业园区集聚效应开始显现，荆州工业新区入园企业达到49家，荆州、沙市、松滋等县市区入园开工项目在15个以上。“双百工程”成效明显，38家重点工业企业销售收入占规模以上工业的59%，楚源年销售收入达到25.3亿元，沙隆达达到18.6亿元，凯乐、四机和吉象、恒隆、天荣、拍马分别超过5亿元和4亿元，纳税过千万元企业达到11家；100个重点项目完成投资29.2亿元，投产和部分投产38个。工业用电量持续较快增长，达到22.5亿千瓦时。招商引资势头良好，签约项目570个（过亿元的项目29个），项目履约率为88.8%；实际到资50.2亿元，增长46.9%。中国化工集团成功收购沙隆达，英国利洁时公司中国总部和生产基地整体迁入荆州开发区，吉象、恒隆、飞利浦、华意、万向、小天鹅、大枫在荆投资规模扩大。农业和农村发展活力进一步增强。深入推进农业结构调整，主要农产品产量增加，粮食增产17.9万吨。水产、畜牧、林业等优势产业快速发展，区域特色逐步显现。农村市场主体建设步伐加快，农业产业化经营水平不断提高。市级以上龙头企业达到93家，新增33家，培育各类农民专业合作组织256个，社会化服务能力增强。加强农村基础设施建设，监利县、公安县、洪湖市和松滋市高产基本农田土地整理项目全面展开，向上争取资金9200多万元。实施“阳光工程”，培训农村劳动力2.6万人次，劳务工资性收入成为农民增收的主要来源。建立健全动物疫病防控监测处置机制，成功扑灭石首天鹅洲高致病性禽流感疫情。城乡市场进一步活跃。以沃尔玛即将入驻北京路为龙头，以库玛整体经营中山路步行街为品牌，以沙隆达广场为中心，荆州正在成为辐射范围更大的区域性商业中心。财政收入实现年初预算目标，结构进一步优化。工商税收占财政收入比重达75.9%，提高9个百分点；烟草销售入库税金1.3亿元，增长38.7%。国税收入突破10亿元大关，达到11.7亿元；地税收入在社保费收入大幅增长的基础上，完成7.7亿元。在政策性减收和增支因素较多的情况下，市级财政较好地完成了年初预算任务，增加对政法系统的财政供给，偿还到期债务6707万元，赤字控制在2000万元以内。金融发展环境进一步改善。在开发银行支持下，组建新的市城投公司，建立土地收购储备、城市建设投资和中小企业担保融资平台。开发银行通过市城投公司，参与重组市商业银行取得实质性进展。扎实推进金融机构不良资产整体打包处置工作，清收国家公职人员拖欠地方金融机构贷款6092万元，完善中小企业信用促进会和担保体系，信用环境建设得到加强。金融机构各项贷款余额256.6亿元，比年初增6.9亿元；各项存款余额439.4亿元，比年初增53.5亿元。市农村信用社、建设银行、农业发展银行、市商业银行积极增加贷款规模，为经济社会发展作出了突出贡献；农业银行、中国银行大力开展优质服务，工商银行努力消化不良债务，有力地支持了地方经济发展。

（二）改革开放取得新进展。农村税费改革继续深化，乡镇综合配套改革在洪湖、监利试点的基础上全面展开，乡镇机关改革任务基本完成；农村土地二轮延包确权发证到户率达95%以上。认真执行国家农业税减免和粮食最低收购价政策，全市减免农业税2.8亿元，发放粮食直补和良种补贴资金1.7亿元；农民因实施粮食最低收购价增加收入9500万元。国有农场综合改革、卫生血防管理体制改革稳步推进。切实加强改制存续企业管理，认真做好非工业企业改革工作，国有资产监管体制初步建立。财政管理体制改革有序推进，部门预算和国库集中支付覆盖面扩大，政府采购规模增加，收支两条线管理得到加强。积极开展农村信用社体制改革，规范发展农村金融服务。深化行政审批制度改革，完善行政服务中心和经济发展环境投诉中心功能，组建市综合招投标中心和采购中心，投资环境进一步改善。外向型经济增势强劲，海关、出入境检验检疫、口岸等工作扎实有效，大通关服务水平提高。外贸出口达到2.4亿美元；盐卡口岸外贸货运量达80万吨，集装箱运量超过2万标箱，分别较上年增长46%和57%；实际利用外资6900万美元，增长15%。

（三）城乡基础设施建设步伐加快。完成全社会固定资产投资116.8亿元。交通建设创历史新高，完成投资20亿元，超过“九五”时期交通投资总和。投资7亿多元的荆襄高速公路荆州段及大桥连接线建成通车，投资30多亿元的荆东高速公路建设进度加快，随岳高速公路、盐卡新港、宦子口船闸和东荆河大桥等重点工程进展顺利，建成通乡公路150公里、通村公路990公里。改造地方铁路设施，运输能力进一步提高。坚持不懈地开展水利建设，堤防整险加固、水库除险及泵站改造有序进行，完成投资2.14亿元、土方5200万方。电力、通信分别完成投资3.4亿元和3.5亿元，电网和通信网络进一步完善。中心城区规划、建设和管理力度加大。建立城区数字地图和数据库，全面开展城市总体规划修编，启动旅游总体规划修编和海子湖旅游目的地规划工作，重点区域控制性详规覆盖率达100%。完成城市基础设施投资1.2亿元，建成玉桥泵站和豉湖渠泵站，改造道路管网和排水设施，困扰多年的渍水问题基本得到解决；完成东方大道、江津路路面铺黑升级20多公里，改造维修北京路非机动车道和红门路、荆沙路等主次干道，城市道路交通条件得到改善；启动护城河、庙湖生态治污工程，着手治理城市水污染问题。加强城市防洪工程建设，护城河护岸工程基本完工，西干渠整治工程进展较快。积极利用国债资金建设城区垃圾发电项目，拓宽城市垃圾处置和综合利用渠道。加强重点片区危旧房改造，适度控制房地产开发规模，引导房地产业健康发展，房地产业完成投资13.7亿元，增长27.8%。理顺城市环卫、监察管理体制，开展以整治占道经营为主的城市环境综合治理工作，城市管理水平进一步提高。县市城区和小城镇加快发展，石首市、松滋市和瞿家湾、调关、新沟、刘家场等镇获全省第三届城镇规划建设管理“楚天杯”奖，监利县周老咀镇被确定为国家历史文化名镇。

（四）各项社会事业全面发展。努力提高科技创新水平。积极增加科技投入，提高科技成果转化能力，新增国家级高新技术企业1家、省级高新技术企业8家，新建楚源合成染料中间体、德炎水产品加工、恒隆汽车转向系统等3个省级工程技术研究中心，争取国家、省级各类科技计划115项，获国家、省科技进步奖24项，公安、洪湖成为全国科技进步先进县市。优先发展教育事业。加强基础教育，积极落实“两免一补”政策，为8万多名义务教育阶段农村贫困家庭学生免去书费和学费，筹资4109万元改造农村中小学校危房10.6万平方米；支持长江大学、荆州职业技术学院和湖北中医药高等专科学校校区建设，推动高等教育、职业教育健康发展。加快发展卫生事业。推进公安、石首新型农村合作医疗试点工作，加强公共卫生服务体系建设，市、县疾病预防控制中心等项目建设基本完成，公共卫生事件应急处置能力明显增强。积极发展文化事业，切实加强文物保护，市文物保护中心成为国家级文物保护科研基地，漆木器脱水技术和丝织品生物保护技术居国内领先水平。社会科学、文学艺术、广播电视、新闻出版、知识产权保护等取得新成绩。广泛开展全民健身活动，成功举办市三运会。扎实做好土地开发、整理、复垦工作，严格土地管理，耕地保护得到加强。认真落实计生政策，全面完成省政府下达的年度责任目标，计划生育率为96.55%。加强社区建设和文明村镇建设，广泛开展群众性精神文明创建活动。深入开展严打整治斗争，社会治安形势基本稳定。扎实推进“平安荆州”创建工作，松滋市被评为全国社会治安综合治理先进县市，成为全省首批“平安县市”。严格落实安全生产责任制，消防、煤矿等重点领域安全生产事故得到有效控制。设立群众接访中心，实行部门联动接访机制，信访集中处置工作扎实有效。村委会换届选举顺利完成，基层民主政权建设进一步巩固。制定依法行政和科学民主决策实施意见，认真实行执法责任制和执法违法责任追究制，加强行政监督和审计监督，积极开展决策咨询和政府新闻发布工作，加强应对重大突发公共事件工作机制建设，政府社会管理能力和工作透明度进一步增强。加强国防教育、国防动员、人民防空、民兵和预备役建设，认真做好军转干部、退伍军人安置和拥军优属工作。妇女儿童和未成年人、老年人、残疾人的合法权益得到保护。圆满完成第一次经济普查任务。气象、地震、档案、地方志、对台、外事侨务、民族宗教、扶贫开发、质量技术监督和无线电管理等工作都有新的进步。

（五）认真解决事关群众切身利益的突出问题。加强社会保障和社会救助体系建设。社会保险扩面新增参保10.5万人次，积极争取国家和省转移支付资金，在提高发放标准的基础上实现“两个确保”；发放农村救灾救助资金787万元，救助14.8万人。坚决纠正损害群众利益的行为，妥善解决群众反映强烈的突出问题，清退教育乱收费资金1201万元、农民负担违规资金837.9万元，兑现拖欠农民工工资5547万元。努力为人民群众办实事，年初承诺的十件实事完成较好。新增城镇就业岗位8.2万个，安置下岗失业人员5.5万人；99家乡镇卫生院改造完成投资2500万元，8个血防综合治理试点工程完成投资4000多万元，免费救治晚血病人3280人；实施大病救助1171人，资助资金329万元，农村“五保”人员集中供养率达到85%；实施“放心菜”工程，中心城区筹资4113.4万元，建成无公害蔬菜生产基地1万亩；“村村通”信息工程完成投资4500万元，发展用户3.9万户；城乡有线电视覆盖工程完成投资5000万元，发展用户10.2万户；边远乡镇客运站场建设完成投资1500万元，实现乡乡通达客运班车；完成五一路等3个片区、5万平方米危房改造任务，启动绿化村等2个片区、8万平方米危房改造工程，减免最低收入者承租国有直管公房租金54.8万元；解决14129名进城务工农民子女就近入学问题；市青少年宫改扩建完成投资550万元，主体工程完工。

市政府及各部门接受人大及其常委会的法律监督和工作监督，接受人民政协的民主监督，认真办理人大代表、政协委员建议、提案、批评和意见。市政府高度重视并认真执行市人大《关于加强洪湖湿地保护的决议》，多次召开政府常务会议专题研究洪湖湿地保护工作，深入实地调查研究、现场办公，研究制定相关政策，组织动员、督促协调拆围工作。在省委、省政府和省有关部门的重视和支持下，组建市洪湖湿地保护局，抓紧制定洪湖湿地综合保护规划和保护区管理办法，积极申报国家级自然保护区和建设项目，认真落实围网拆除补偿政策，稳步推进拆围和渔民安置工作。在拆围工作中，洪湖周边广大干部群众顾全大局，积极支持湿地保护工作；监利县、洪湖市及沿湖乡镇成立专班，实行责任制管理，完成拆围面积15万亩，发放补偿和安置资金2701万元，预计三年拆围任务可提前一年完成。

各位代表，“十五”时期和2005年，我市改革开放和现代化建设取得了显著成就，这些成绩来之不易，是全市人民团结奋斗、真抓实干的结果。在此，我谨代表市人民政府，向为荆州改革发展稳定作出积极贡献的全市广大干部群众，向人大代表、政协委员、各民主党派、工商联、人民团体和无党派爱国人士，向来荆投资的海内外人士，向在荆的中省垂直管理机关、企事业单位干部职工，向人民解放军、武警官兵和政法干警，向所有关心、支持荆州建设与发展的各界朋友，致以崇高的敬意和衷心的感谢!

在看到成绩的同时，我们也清醒认识到，“十五”部分经济指标未能达到预期要求，发展不快、发展不够仍然是荆州面临的主要问题，政府工作还有不少薄弱环节，经济社会生活中还存在一些突出的矛盾和困难。一是制约经济发展的深层次问题尚未从根本上得到解决，体制改革、结构调整、自主创新、转变经济增长方式等方面的工作任务繁重，特别是改善信用环境、缓解财政收支矛盾的难度较大。二是农业基础仍很薄弱，乡村债务包袱沉重，农民组织化程度较低，农产品市场竞争力不强，农田水利设施老化，农村公共事业发展滞后，农民持续增收的难度大。三是工业经济总量不大，产业聚集度不够，县域经济、民营经济活力不足，工业运行质量有待提高。四是中小企业融资困难，要素瓶颈制约明显，经济发展环境有待进一步改善。五是就业再就业压力较大，社会治安、城市管理和环境保护不尽人意，影响社会稳定的诸多因素仍很突出。六是政府机关工作中存在官僚主义和形式主义以及腐败现象，政府自身建设还需进一步加强，等等。在今后的工作中，我们将高度重视，坚持不懈地采取有效措施认真加以解决。

二、“十一五”时期经济社会发展的总体要求和主要任务

未来五年，是荆州抢抓机遇、乘势而上、加快发展的关键时期。在中共荆州市委《关于制定国民经济和社会发展第十一个五年规划的建议》指导下，市政府经过深入调查研究，广泛征求各方面意见，制定了《纲要(草案)》。《纲要(草案)》分析了“十一五”时期荆州经济社会发展面临的环境和条件。一是经济发展环境趋好。全球经济处于新一轮增长周期，国内经济平稳较快增长，湖北经济发展步伐加快，为荆州“十一五”发展提供了良好的外部环境。二是政策扶持力度加大。随着中部崛起战略的深入实施，国家将在产业发展、资金投入、重大基础设施建设等方面加大对中部地区的扶持力度，增加对“三农”的投入，加快新农村建设步伐，为荆州加快发展提供了重大政策机遇。三是自身基础明显改善。随着长江干堤全面加固和取消农业税，长期困扰我们的防汛任务重、农民负担重等矛盾和问题有较大缓解。通过实施“工业兴市”战略，自我发展能力和综合经济实力增强。特别令人鼓舞的是，沪汉蓉高速铁路客运专线武汉-荆州-宜昌段和荆岳长江大桥年内将开工兴建，荆东高速公路将在今年十月建成通车，随岳高速公路、汉南至监利高速公路和监利至荆州一级公路正在抓紧建设，盐卡港可望升级为国家一类口岸，引江济汉工程、松滋至石首一级公路建设前期工作有效展开，荆岳铁路已经纳入国家铁路网络建设规划，必将极大地改善荆州交通状况，提升区位优势，为经济社会发展注入新的活力。四是荆州人民期盼加快发展、再创辉煌的强烈愿望，将成为推动经济社会发展的强大精神动力。未来五年，我们完全有条件发展得更快一些、更好一些。

基于上述分析和判断，《纲要(草案)》提出了“十一五”时期经济和社会发展的总体要求和发展目标。总体要求是：以邓小平理论和“三个代表”重要思想为指导，坚持用科学发展观统领经济社会发展全局，深入实施“工业兴市”战略，加快推进社会主义新农村建设，着力转变经济增长方式、提高自主创新能力、深化改革开放、完善体制机制，着力推进新型工业化、农业产业化、城镇化进程，着力加快“新荆州、古荆州、美荆州、大荆州”建设步伐，全面增强综合实力、发展活力和区域竞争力，把荆州建设成为江汉平原的中心城市。

主要发展目标是：力争全市生产总值年均增长10%以上，提前实现比2000年翻一番；全社会固定资产投资年均增长18%，五年累计努力达到1000亿元；社会消费品零售总额年均增长13%，外贸出口年均增长15%，财政收入年均增长9%，城镇居民人均可支配收入年均增长8%，农民人均纯收入年均增长6%，人口自然增长率控制在6‰以内，城镇登记失业率控制在4.5%以内，万元生产总值综合能耗下降20%左右。需要说明的是，“十一五”规划是政策性引导市场配置资源的规划，不是指令性项目建设计划，在执行过程中将按照市场需求进行调整，是一个动态的、不断调整的规划。上述发展目标中，生产总值、固定资产投资等是预期性的，人口自然增长率、万元生产总值综合能耗等是约束性的，提出这些目标，是确保加快荆州新一轮经济发展的需要，务必努力完成。

为实现上述目标，未来五年，要坚持“十五”时期形成的发展思路和工作方法，始终用科学发展观统领经济社会发展全局，做到“五个坚持”，即：坚持加快发展、科学发展，突出实施“工业兴市”战略，增强自主创新能力，推进经济结构战略性调整，转变经济增长方式，努力实现一、二、三产业协调发展；坚持统筹城乡发展，加快社会主义新农村建设步伐，增强城市集聚辐射带动能力，努力实现城乡协调发展；坚持深化改革开放，积极培育市场主体，突破性发展民营经济，努力形成充满活力的体制机制；坚持以人为本，认真解决经济社会发展中的突出矛盾和关系群众切身利益的突出问题，不断提高人民生活水平和质量，努力构建“和谐荆州”；坚持依法行政，切实转变政府职能，不断提高驾驭经济社会发展全局的能力和水平，努力建设服务型政府。主要任务是：

（一）强力推进新型工业化。加快工业化进程，“工业兴市”是主战略，开发区、园区是主战场。我市与发达地区的差距拉大，主要是由于工业整体实力不强，工业化水平偏低。必须紧紧围绕把荆州建设成为江汉平原的新型工业城市，更新发展观念，调整发展思路，转变发展模式，增强自主创新能力，积极发展循环经济，节约利用能源资源，整体提升工业竞争力，加快新型工业化进程，力争“十一五”期间工业增加值年均增长13%。依托现有产业基础，瞄准未来产业发展方向，重点建设汽车零部件、纺织、精细化工、食品加工、森工造纸等生产基地，培育和扶持电子信息、生物技术与新医药、新材料等高新技术产业。力争“十一五”期间，机械、轻工业增加值年均增长13%以上，纺织、化学和医药工业年均增长12%以上，高新技术产业增加值年均增长20%以上。鼓励各类要素向优势行业和优势企业集中，积极推动产业集群、企业集聚、集约发展。引导中小企业向“专、精、特、新”方向发展，增强产业配套能力。坚持把招商引资作为战略性举措来抓，积极引进大项目、大企业，不断壮大工业实力和规模。加强工业园区建设，形成以荆州工业新区和县市区工业园为主体的产业集聚平台。大力培植精品名牌，力争“十一五”期间创中国名牌产品1-3个。

（二）努力建设社会主义新农村。建设社会主义新农村，是新时期做好“三农”工作的新思路、新要求、新举措。荆州是农业大市，社会主义新农村建设任务重大而艰巨。按照“生产发展、生活宽裕、乡风文明、村容整洁、管理民主”的总体要求，以稳定和完善家庭承包经营制度为基础，以发展农村经济和增加农民收入为中心，以科学规划为龙头，以增加投入为保障，以项目建设为载体，有计划、分步骤地推进社会主义新农村建设，力争“十一五”期间农业增加值年均增长3.5%。加强农业基础设施建设，完善防洪和排灌工程体系，推进农业综合开发和高产农田项目建设，提高农业综合生产能力。在稳定粮食生产的基础上，扩大优质粮棉油产量，突出发展水产、畜牧、林业等优势产业，调整优化农业经济结构，培育壮大龙头企业，积极发展农民专业合作组织，推进农业产业化经营，健全农业技术推广、农业信息服务、农产品质量安全和动植物病虫害防控体系，加快发展现代农业。全面发展农村公共事业，增加对农村教育、卫生、文化、计划生育等方面的投入，加快农村公路、通信、广播电视、安全饮水和生态家园工程等建设步伐，加大扶贫开发力度，改善农村生产生活条件。深入推进以乡镇机构、农村义务教育和县乡财政体制改革为主要内容的农村综合改革，建立促进现代农业发展、增加农民收入的长效机制。广开增收渠道，充分挖掘农业内部增收潜力，加快发展农村二、三产业，继续推进农村劳动力转移，千方百计增加农民收入。

（三）积极发展现代服务业。服务业是现代经济社会发展的重要支撑，是提升城市综合竞争力的内在要求。要紧紧围绕把荆州建成商贸物流中心城市和历史文化旅游目的地，大力培育新型服务业，全面提升传统服务业，引导各类服务业向产业化、社会化方向发展，不断提高服务业的比重，力争“十一五”期间第三产业增加值年均增长12%。坚持把文化旅游产业作为支柱产业来培育，作为战略产业来扶持，突出楚文化、三国文化和生态文化特色，用产业化的办法、市场化的思路，打破部门、行业、区域、所有制界限，整合文化旅游资源，形成有利于文化旅游产业加快发展的体制机制，形成以荆州历史文化景区为核心、以洪湖渔家生态景区、洈水风景区、长江天鹅洲湿地景区为支撑的旅游发展格局。完善文化产业政策，培育壮大文化市场主体，促进文化产业快速发展。充分发挥资源、区位、市场优势，突破性发展现代物流业，支持物流园区和配送系统建设，培育壮大骨干物流企业，推动农产品、农资、纺织等专业物流发展。运用现代经营方式和信息技术，改造提升传统商贸流通业，大力发展金融保险、信息咨询、中介服务等服务业，积极发展市场潜力大的娱乐休闲、社区服务、职业培训等服务业。

（四）着力提高城市化水平。加快城市发展步伐，提高城市化水平，是推进经济社会结构转型的重要战略任务。坚持从实际出发，加快完善交通、电力、通信等现代基础设施，推进以中心城区为龙头、县（市）城区为支撑、中心城镇为依托、建制镇为基础的城镇体系建设，提高集聚辐射带动能力，加快城市化进程。加强综合交通运输体系建设，规划投资244亿元。以“三横四纵三桥”(三横：沪蓉高速公路荆州段、汉南至监利高速公路和监利至荆州一级公路、松滋至石首一级公路；四纵：荆东高速公路、随岳高速公路荆州段、仙桃至洪湖一级公路、潜江至石首一级公路；三桥：荆州长江大桥、荆岳长江大桥、洪湖东荆河大桥)和盐卡港、江汉航道网为骨架，以乡村公路网络为依托，以沪汉蓉高速铁路客运专线荆州段开工建设和荆岳铁路开展前期工作为契机，以重要站场、物流园区建设为平台，经过五到十年的努力，把荆州建设成为区域性交通枢纽、长江中游重要港口、国家一类口岸，在江汉平原率先实现交通现代化。进一步加快电网建设步伐，确保电力建设与城乡发展相协调，能源规划投资160亿元。加强通信网络建设，建设高效、安全的现代信息基础设施，规划投资17亿元。突出中心城区发展，重点建设“两区两城”。即：以东方大道为核心建设14平方公里的荆州工业新区，以北京路为核心建设商业中心区，以荆州古城改造为核心建设三国主题公园，以纪南城遗址公园为核心建设海子湖旅游目的地，加快环城公路网规划和建设，形成富有特色的产业布局和城市空间布局。力争到“十一五”期末，全市城市化率达到42%，中心城区建成区面积达到68平方公里，人口达到75万人。

（五）加快改革开放步伐。改革开放是实现经济社会又快又好发展的强大动力。必须深化政府、企业、市场和社会领域的改革，为加快发展提供体制保障。加快政府管理体制改革，切实转变政府职能，更加注重社会管理和公共服务，健全科学民主决策机制和行政监督机制，推行政府绩效考评制度，建设服务型政府。深化国有企业改革，完善法人治理结构，加快建立规范的现代企业制度。完善国有资产监管体制和出资人制度，促进国有资产保值增值。努力消除非公有制经济发展的体制性、政策性障碍，引导个体、私营企业制度创新，促进民营经济快速发展。培育土地、技术、资本和劳动力等要素市场，完善现代市场体系。深化财税体制改革，建设公共财政体系。分类推进事业单位改革，精简机构，提高效能，强化服务，建立新型的管理体制和运行机制。坚持以全方位开放促改革、促发展，转变外贸增长方式，扩大对外贸易规模，拓宽利用外资领域，提高利用外资质量和水平，力争“十一五”期末外贸出口总额和利用外资总量比“十五”期末翻一番。

（六）加强自主创新能力建设。自主创新是调整产业结构、提升经济竞争力的中心环节。必须围绕建设创新型城市，充分利用现有产业基础和科教优势，整合科技资源，提高科技成果消化吸收转化能力，切实把经济社会发展转到依靠科技进步和提高劳动者素质的轨道上来。深化科技管理体制改革，落实科技创新扶持政策，形成科技不断促进经济社会发展、社会不断增加科技投入的良好机制。加强以企业为主体、以市场为导向、产学研相结合的技术创新体系建设，支持一批龙头企业建立国家级和省级工程技术研究中心。培育和发展各类科技中介服务机构，抓好科技创新公共服务平台建设。在汽车零部件、机械装备、精细化工、生物医药等重点产业领域，组织实施一批重大科技专项，力争突破一批关键技术。支持在荆高校加强优势学科建设，发挥其在技术创新中的骨干和引领作用。加大引进人才、引进智力工作力度，广泛吸纳和集聚各类优秀人才。加强企业经营管理人才、专业技术人才和农村适用人才队伍建设，营造“爱才、育才、聚才、用才”的良好社会氛围。

（七）大力发展各项社会事业。协调发展各项社会事业，加强和谐社会建设，是推进经济社会发展的重要保障。必须坚持把就业放在更加突出的位置，统筹城乡就业，努力扩大就业规模。依法扩大社保覆盖面，完善社会保障体系。建立和完善特困群众社会救助体系，重视解决城乡困难群众的生产生活问题。突出教育优先发展战略地位，巩固和提高义务教育水平，推进各类教育改革与发展。积极扩大新型农村合作医疗试点，力争2008年基本普及；抓好城市社区卫生服务工作，切实加强血吸虫病、艾滋病等重大疾病防治，完善疾病防控、应急处理、医疗救治和卫生监督体系。大力发展文化体育事业，广泛开展群众性精神文明创建活动。积极做好信访工作，努力化解人民内部矛盾。加强社会治安综合治理，切实维护社会稳定。加强国防动员和民兵预备役工作，争创全国“双拥”模范城。坚持经济社会发展与人口资源环境相协调，严格控制人口增长，加强基本农田保护，突出抓好洪湖、长湖、洈水等重点水域生态环境整治和资源保护，促进可持续发展。

三、2006年工作建议

今年是实施“十一五”规划的起步之年，做好政府各项工作，意义十分重大。全市经济社会发展的主要预期目标是：生产总值增长10%以上，全社会固定资产投资增长18%左右，社会消费品零售总额增长12%，外贸出口增长15%，利用外资增长10%，万元生产总值综合能耗下降4%，财政收入增长8%左右，农民人均纯收入增加160元，城镇居民人均可支配收入增长8%，人口自然增长率控制在5.5‰以内。为实现上述目标，重点做好以下八个方面的工作。

(一)以实施“工业兴市”战略为主线，不断提高经济发展质量和效益。进一步优化工业结构，转变经济增长方式，不断提高工业整体素质，确保全市规模以上工业增加值增长18%。加快推进产业集群。重点抓好汽车零部件、纺织、精细化工、水工环保和石油机械制造、家电、食品加工、森工造纸等优势产业调整与发展，积极延伸产业链，提高综合配套能力和产业集中度。积极扶持信息产品制造、新材料、生物医药等产业发展，提升高新技术产业比重。引导企业增加研发投入，提高自主创新能力。积极推行清洁生产，大力发展循环经济，提高资源综合利用水平。积极争创精品名牌，支持企业与国际标准认证体系接轨，扩大与知名品牌合作，提高产品竞争力。加快实施“双百”工程。对全市100家重点骨干企业，实行分类指导，力争今年楚源销售收入过30亿元，沙隆达过20亿元，一批企业过5亿元。对发展前景好的中小企业，加强扶持引导，促其上规模、上水平，力争新增规模企业40家。对100个投资过千万元的重点项目，加强跟踪服务，抓好沙市热电厂60万千瓦热电联产、凯乐短纤、亿钧自洁玻璃、城区垃圾发电、大田18万吨合成铵和30万吨尿素、711船舶自动识别系统和生产线改造、洪城大型电站系列球阀、沙隆达吡虫啉和毒死蜱及离子膜碱、奥达高档服装面料、菲菲律印染生产线等43个投资过亿元的项目建设，力争工业固定资产投资增长20%以上。对税收过3000万元的企业，从政策、融资等方面给予重点扶持。加快工业园区建设。加大投入力度，加强基础设施建设，规划和建设好各级各类经济开发区和工业园区。加强重大设备共享平台和产业共性技术开发服务平台建设，完善金融服务、人才培训等配套功能，提高园区管理和服务水平。加大项目引进和建设力度，吸纳一批产业关联度强的企业，形成特色园区。荆州工业新区力争新引进项目25个，新增投产企业10家；各县市区开发区、工业园区都要力争新引进项目10个，新增投产企业5家。加快发展民营经济。认真贯彻落实国家和省鼓励、支持和引导个体私营等非公有制经济发展的各项政策措施，坚持走县域经济“一主三化”发展路子，按照“非禁即入”、公平待遇原则，鼓励非公有资本进入基础设施、公用事业和社会事业等领域，参与公有制经济的改制改组。加强对民营企业创业辅导、融资担保、技术创新、人才培训等服务，提高民营企业整体素质，支持有条件的企业做大做强，使一批民营企业成为经济发展的排头兵。鼓励自主创业，让民间资金更多地转化为生产经营资本，扩大民间投资。加快培育企业家队伍。高度重视企业家的培养、引进和使用，尊重企业家，尊重纳税人，尊重创业者，激励各类企业经营管理人员在“工业兴市”中建功立业、多做贡献。

(二)以建设社会主义新农村为目标，切实做好“三农”工作。坚持把解决“三农”问题作为重中之重，认真落实各项农村政策，调动农民积极性和创造性，推动社会主义新农村建设，力争全市农业增加值增长5%。切实抓好农业产业化经营。大力发展具有竞争优势的优质粮棉油、畜禽、水产、果蔬、林业等产业，推进专业小区和板块建设，推进标准化、规模化生产，提高优质农产品比重。在稳步发展粮棉油生产的基础上，力争水产品产量增2万吨、生猪出栏增30万头，家禽出笼增500万只，畜牧养殖小区增15个。下大力气做大做强龙头企业，突破性发展农民专业合作组织，不断提高产业化经营水平。大力实施“阳光工程”，加强农村劳动力转移输出，促进打工经济、劳务经济发展。切实抓好农村基础设施建设。重点抓好荆江河势控制、荆江和洪湖分蓄洪区建设、荆南四河和四湖流域综合治理、洈水水库除险加固等工程项目，积极改造大型泵站、大中型灌区，加快水稻主产县市高产农田项目建设步伐。大力开展农村道路、电网、通信、广播电视、安全饮水、沼气等基础设施项目建设，积极争取省政府在荆州实施血防整县推进工程。扎实做好防汛抗灾工作，确保农业增效、农民增收。切实抓好农村公共事业发展。开展农业科技培训示范，推进农业科技进步。调整农村中小学校布局，提高农村办学质量和水平。加强农村公共卫生和医疗服务体系建设，积极推广新型农村合作医疗制度，在巩固公安和石首试点成果的基础上，增加松滋和监利试点，落实参合农民财政补助标准，由每人每年10元增加到15元。高度重视动物防疫检疫，认真做好高致病性禽流感等重大疫病防控工作。切实抓好农村综合改革。进一步深化乡镇综合配套改革，按照竞聘上岗、目标责任、合同管理的要求，积极推进农村水利、农业技术推广、畜禽防疫等事业单位改革，增强农村发展活力。落实各项农村政策，规范农村土地流转，完善“一事一议”和村务公开制度，加强农村财务管理，积极化解乡村债务，努力减轻农民负担。切实抓好试点示范。坚持规划先行、试点先行，注重从实际出发，尊重农民意愿，因地制宜，分类指导，有计划有重点地推进社会主义新农村建设。加强村镇建设和农村居民点建设的规划指导，重点改善农民生产设施条件和生活环境，不盲目攀比，不强求一律，不加重农民负担。市政府将在公安县花基台血防综合治理和荆州区李埠镇“放心菜”工程建设的基础上，推进社会主义新农村建设试点工作。各县市区都要办好试点，逐步推开。

（三）以文化旅游和现代物流为突破口，积极发展现代服务业。做大做强文化旅游业。加强以三国文化、楚文化和生态文化为主题的景点景区开发，加强宣传和推介工作，不断提升文化旅游业发展水平。突出抓好海子湖旅游目的地道路、供水、供电等基础设施建设，招商开发酒店、度假村等旅游设施。认真组织开展熊家冢古墓发掘工作，加强市文物保护中心和中国漆木器博物馆建设，开工建设纪南城遗址公园。抓紧启动石首天鹅洲湿地生态旅游景区建设项目，加强洪湖、洈水旅游基础设施建设。加快发展现代物流业和商贸流通业。支持长江物流园、小北门物流园等物流园区建设，加快荆州现代物流中心、农产品物流交易中心和轻纺市场等物流市场建设，促进专业物流发展。加强商业网点规划与管理，推进商业结构和区域布局调整，加快经营业态创新。积极创造条件，让沃尔玛顺利入驻荆州，带动以北京路为重点的中心商圈建设。积极推进流通体制改革试点工作，实施“万村千乡”市场工程，扩大农村市场消费。坚持扶持与规范并举，促进信息服务业、社区服务业、中介服务业等现代服务业健康发展。

（四）以完善城市功能和美化城市环境为重点，加强城乡建设和管理。加快中心城区发展。在现有城市骨架的基础上，完善城市基础设施，提高城市发展内涵和质量。坚持科学规划，拓展规划编制深度，严格规划管理，提高规划的前瞻性、科学性和权威性。抓好《荆州市城市总体规划》修编，完成荆州古城保护和纪南城遗址保护规划编制，优化城市布局，突出城市特色。全面推行“四线”规划管理，加强对城区湖面、水体的保护。加快沿江大道和荆沙大道建设，改造南湖路、长港路和航空路，抓好城区主次干道的绿化配套工作。启动红光污水处理系统，继续抓好护城河疏浚整治和庙湖治理工程，配套燎原路、月堤路（江津路至沙岑段）和城区部分管网，努力解决水污染问题。继续抓好西干渠整治工程，开工建设长湖围堤整治工程，不断增强城市防洪能力。进一步做大做强市城投公司，充分利用开发银行的融资优势，积极筹措建设资金，提高政府土地收益和城市经营水平。加大城市管理力度，积极推行相对集中行政处罚权，完善“门前三包”管理制度，突出整治占道经营和“脏乱差”，努力改善市容市貌。加强县市城区和中心镇建设。加强规划，突出重点，合理布局，提高县市城区和中心镇产业集聚和人口吸纳能力。多渠道筹集建设资金，抓好基础设施、公共服务设施建设，不断完善和提升城镇综合功能。加强环境卫生、交通秩序管理，努力提高城镇管理水平。认真落实促进县域经济发展的政策措施，支持县市培育竞争力强的产品和产业集群，以产业支撑带动城镇发展，不断壮大县域经济实力。加强基础设施项目建设。抓好荆东高速公路、随岳高速公路、荆岳长江大桥、洪湖东荆河大桥、盐卡港综合码头扩建工程、输变电工程等重点项目建设，切实做好沪汉蓉高速铁路客运专线荆州段、引江济汉工程开工的各项准备工作，努力争取荆岳铁路提前建设。

（五）以改革开放为动力，增强经济社会发展活力。进一步深化各项改革。继续推进国有企业改革，妥善解决职工安置等遗留问题，规范法人治理结构，建立健全现代企业制度。加强国有资产管理，增加国有资产收益。抓好市直部门与所办企业、经济实体脱钩改制和企业化管理的事业单位改革改制工作。支持上市公司规范发展，积极培植上市后备企业。深化部门预算、国库集中收付、政府采购和收支两条线等财政改革，推行县市区财政与编制公开，提高财政管理水平。加强现代市场体系建设，重点培育发展劳动力、技术、土地等要素市场。进一步整顿和规范市场经济秩序，严格市场准入，抓好食品、药品、烟草、农资等市场专项整治。加大招商引资工作力度。继续实行主要领导用主要精力抓招商引资和主要领导驻点招商制度，落实招商引资目标责任。把招大项目作为招商工作的重中之重，狠抓大项目的策划、编制和引进。积极推行小分队招商、专题招商、产业链招商，突出企业招商主体地位，精心组织长三角、珠三角和国外、境外重大招商活动，切实提高招商效果。认真抓好签约项目落实工作，力争客方实际到资增长30%。努力提高对外开放水平。鼓励和引导外商采取合资、独资、合作和并购、增资等形式开展投资，努力提高利用外资的质量和水平。扩大化工、机电、轻纺、农产品等出口规模，保持外贸出口持续快速增长势头。支持洪城、恒隆等有条件的企业加入跨国公司全球采购体系、营销体系和供应链体系，带动技术和劳务出口。

（六）以培育自我发展能力为核心，扎实做好财税金融工作。认真做好财税工作。坚持依法治税、依率计征，加强收入组织协调，严格重点税种、税源管理，确保财政收入稳定增长。优化支出结构，确保工资发放、社会保障投入、机关正常运转和法定支出。设立中小企业和信息产业发展基金，合理整合财政专项资金，提高支工支农资金使用效益。严肃财政纪律，开展财政支出绩效评价，规范财政性资金管理。加强政府债务管理，严格控制新增债务，积极化解财政困难。逐步理顺市区财税管理体制，调动各方面聚财、理财积极性。充分发挥金融对经济社会发展的支持作用。重视和支持人民银行加大货币政策指导力度，积极推动国有商业银行和农业发展银行健康发展，鼓励保险、证券等金融机构拓展业务，扶持地方金融机构做大做强。支持农村信用社改革，完成好县市联社的统一法人改革任务。深入开展与开发银行的合作，实现县市全覆盖。加强中小企业信用担保工作，多形式扩大担保公司规模，努力破解中小企业融资难问题。认真做好不良资产打包处置工作，积极清收公职人员所欠贷款，加强金融监管和信用体系建设，努力营造良好的信用环境。制定考核奖励政策，鼓励金融机构增加信贷投入。积极引导信贷资金投向城乡基础设施建设和工农业发展等重点领域和薄弱环节，扶持外向型经济、产业集群、高新技术产业和文化旅游产业发展，推动招商引资和经济结构调整。

(七)以建设和谐社会为主题，妥善解决事关民生的热点难点问题。做好就业再就业和社会保障工作。认真落实就业再就业优惠政策，将再就业优惠证发放范围扩大到厂办集体企业下岗职工，鼓励自谋职业和灵活就业。加强就业培训和社区职介服务，对城镇零就业家庭、农村零转移农户实施就业援助。依法扩大社会保障覆盖面，加大社保费征缴力度，认真解决进城务工人员、灵活就业人员的社会保障问题。高度关注弱势群体和困难群众生活中存在的现实问题，切实做好城镇低保户、农村困难户救济救助工作，帮助解决子女入学、就医等实际困难。全面发展各项社会事业。深入开展精神文明创建活动，提高城乡文明程度。增加科技投入，积极开展企业技术创新示范工作。积极整合教育资源，全面推进素质教育，提高义务教育质量。大力发展高等教育和职业教育，积极支持长江大学建设成为高水平综合性高校，支持荆州职业技术学院建设成为国家示范性职业技术学院，支持湖北中医药高等专科学校做大做强。深化医疗服务体制改革，抓好血吸虫病、艾滋病等重大疾病防治，提高城乡医疗卫生水平。认真实施省政府血防整县推进工程，积极向上争取血防综合治理项目，抓好8个血防综合治理试点验收工作，总结推广试点工作经验，巩固和扩大血防综合治理成果。加强对医疗服务机构的监督和管理，规范医疗服务价格，逐步解决群众看病难、看病贵问题。加强体育设施建设，大力发展体育事业。加大控制出生人口性别比和流动人口管理力度，稳定低生育水平。严格土地管理，保护耕地资源。加强对重点污染源的整治，维护生态平衡。继续抓好洪湖湿地保护工作，力争今年完成围网拆除任务。认真做好第二次农业经济普查工作。加强民主法制建设。自觉接受人大及其常委会的法律监督、政协的民主监督和新闻舆论监督，认真办理人大代表建议和政协委员提案。加强与各民主党派、工商联、无党派人士的联系，充分发挥工会、共青团、妇联、社科联、文联、残联等社会团体的作用。加强社区建设，健全基层群众自治组织和民主管理制度，提高社区管理和服务水平。坚持群众来访联合接待制度，规范部门信访联动机制，重点解决农村征地、城镇拆迁、企业改制、企业违法排污过程中损害群众权益，以及拖欠农民工工资、教育和医疗领域乱收费等问题。积极开展法律援助，依法保障弱势群体的合法权益。加强社会治安综合治理，做好国家安全工作，依法打击各种犯罪活动。加强安全生产监督管理，防止重特大安全事故发生。建立健全社会应急救援和社会动员机制，提高处置突发性事件的能力。继续加强国防教育、国防动员、民兵、预备役建设和人防工作。

(八)以提高行政能力为着力点，切实加强政府自身建设。坚持依法行政。加强政府法制工作，积极推进相对集中行政处罚权和综合执法试点，规范行政审批和执法行为，解决职能交叉、多头执法等问题。认真落实行政执法责任制度和过错追究制度，加大执法监督力度，促进公正执法、文明执法。切实转变政府职能，推进政府管理创新，在继续做好经济调节和市场监管的同时，更加注重履行社会管理和公共服务的职能。坚持科学决策。加强对重大经济社会问题的调查研究，积极发挥市政府咨询委员会的作用，完善公众参与、专家咨询和政府决策相结合的行政决策机制，健全重大决策的规则和程序，实行依法决策、科学决策和民主决策，不断提高行政决策水平。善于运用市场经济的办法和民主与法制的手段解决实际工作中的矛盾和问题，创造性的开展工作，不断提高行政能力和水平。加强电子政务建设，完善新闻发布制度，推进政务公开，提高政府工作的透明度和公众参与度。坚持勤政廉政。牢固树立科学发展观、正确的政绩观，发扬脚踏实地、埋头苦干的工作作风，讲实话，重实干，办实事，求实效，坚决克服形式主义和官僚主义。认真办好各级行政服务中心、经济发展环境投诉中心、综合招投标中心、政府采购中心，完善工作机制，不断提高服务质量和水平。坚持以硬措施整治软环境，重点整治办事效率低、服务态度差、推诿扯皮和“吃拿卡要”等问题，进一步优化发展环境。加强廉政建设，强化行政监督和审计监督，加强领导干部任期责任审计，深入开展行风评议工作，认真纠正损害群众利益的不正之风，坚决查处各种腐败行为，树立人民政府的良好形象。坚持执政为民。始终把改善民生放在政府工作的突出位置，按照量力而行、尽力而为的原则，突出抓好城乡群众最为关心的就业、教育、医疗、住房等问题，重点办好十件实事。 1、千方百计扩大就业和再就业，新增城镇就业人员7.5万人，安置下岗失业人员再就业4.6万人，对“零就业家庭”中有能力、有愿望的劳动者实施就业援助，确保就业一人；2、加强农村劳动力转移培训工作，示范性培训(技能培训)5万人，新增外出务工农民10万人；3、继续搞好血防综合治理工程，免费救治晚血病人3000人，争取省政府血防整县推进工程在我市重疫区实施；4、继续开展城乡贫困群众大病医疗救助工作，救助起付线由2000元降为500元，大病救助种类由5类增加到8类；5、积极做好新型农村合作医疗试点工作，新增参合农民120万人；6、继续实施“放心菜”工程，建立健全市县两级蔬菜质量安全监测体系，扩大“放心菜”专供点覆盖面；7、加大城区水环境治理力度，启动运行红光污水处理系统，完成护城河和庙湖水体治理工程；8、建设200套廉租房，解决低保家庭中的无房户和特殊困难家庭的住房问题，对承租公有住房的低保家庭继续实行租金减免；9、加强农村通信设施建设，投资5000万元，实施“乡镇通宽带”工程；10、积极改善农村交通条件，改造县乡公路100公里，修建通村等级公路800公里，改造达标乡村渡口100个，新建农村公路候车亭50个。

各位代表，回顾“十五”，全市人民创造了令人瞩目的成就，我们为此感到自豪；展望“十一五”，未来发展的宏伟蓝图催人奋进，我们更加充满信心。让我们紧密地团结在以胡锦涛同志为总书记的党中央周围，在省委、省政府和市委的正确领导下，解放思想，锐意进取，团结一致，扎实工作，为开创荆州更加美好的明天而努力奋斗!
